# Barriers and pathways to environmental surveillance of antibiotic resistance in middle- and low-income settings: a qualitative exploratory key expert study

**DOI:** 10.1080/16549716.2024.2343318

**Published:** 2024-05-30

**Authors:** Ann-Christin Peters, D. G. Joakim Larsson, Ramanan Laxminarayan, Christian Munthe

**Affiliations:** a Department of Philosophy, Linguistics and Theory of Science, University of Gothenburg, Gothenburg, Sweden; b Centre for Antibiotic Resistance Research in Gothenburg (CARe), Gothenburg, Sweden; c Department of Infectious Diseases, Institute for Biomedicine, University of Gothenburg, Gothenburg, Sweden; d One Health Trust, Washington, DC, USA; e One Health Trust, Bangalore, India; f High Meadows Environmental Institute, Princeton University, Princeton, NJ, USA

**Keywords:** Antimicrobial resistance, environmental health, global health, one health, research policy, public health

## Abstract

**Background:**

Local and global surveillance of antibiotic resistance (ABR) has proven a challenge to implement effectively in low- and middleincome (LMI) settings. Environmental surveillance solutions are increasingly highlighted as a strategy to help overcome such problems, and thus to promote global health as well as the local management of ABR in LMI countries. While technical and scientific aspects of such solutions are being probed continuously, no study has investigated their practical feasibility.

**Objective:**

Explore practical barriers for environmental surveillance of ABR in LMI countries, and pathways for surveillance experts to manage these.

**Methods:**

To start charting this unknown territory, we conducted an explorative, qualitative interview study with key informants, applying a constructivist grounded theory approach to analyze the results.

**Results:**

Barriers were identified across infrastructural, institutional and social dimensions, and pathways to manage them were mostly counterproductive from an ABR management perspective, including avoiding entire regions, applying substandard methods and failing to include local collaborators.

**Conclusion:**

The research community as well as international agencies, organizations and states have key roles and responsibilities for improving the prospects of feasible environmental ABR surveillance in LMI-settings.

## PAPER CONTEXT

- **Main result:** Physical infrastructural, institutional and socio-cultural barriers to environmental antibiotic resistance surveillance in low- and middle-income countries contribute to global health injustices as well as ineffective global antibiotic resistance management, while surveillance experts report pathways to manage these barriers which tend to worsen these negative effects.

- **Added value:** This seminal study is the first to probe this area, and thus provides unique new insights for further research and improved practices, as well as practical suggestions for research and policy to this effect.

- **Practical implications:** As environmental strategies are main candidates to avoid known problems to achieve effective antibiotic resistance surveillance in low- and middle income-settings, causing major gaps in global health surveillance as well as problems to clinically manage resistant infections in local health systems, it is important that the implementation of environmental surveillance strategies are optimal and avoid counterproductive measures to overcome obstacles.

## Background

Antimicrobial resistance (AMR), particularly antibiotic resistance (ABR), constitutes a significant threat to global health and modern healthcare [1,2]. Surveillance and monitoring of ABR is a cornerstone in managing resistance [3,4], which is also emphasized in the World Health Organization’s (WHO) Global Action Plan on AMR [5]. The global AMR surveillance system, GLASS, was created as the clinical surveillance component of this plan in 2015. Its purpose is to serve as a platform to develop global surveillance standards and create estimations of the global burden of AMR generally, and ABR in particular [6]. However, the surveillance of ABR in low- and middle-income (LMI) countries is challenging due to non-existing or weak surveillance systems [7], and many such countries are still absent from the GLASS collaboration or lack reliable surveillance data. Besides sparse data on prevalence, incidence and geographical distribution, there is selection bias based on unequal access to health services about who is tested for ABR and what data are entered into laboratory systems. Furthermore, data on systematic quantification of antibiotic drug usage are lacking [8]. This prevents the GLASS system from painting a representative picture and undermines local measures to curb ABR.

The underdeveloped ABR surveillance in LMI countries undermines effective global and local ABR management and creates a problem of global inequality related to the surveillance objectives indicated at the outset by negatively influencing both basic knowledge production (e.g. regarding the evolution of ABR), regional and local risk assessment and ABR management in LMI-settings. Consequently, a problem of epistemic injustice [9] for LMI-countries in global ABR management emerges and enhances other global health inequities [10].

It is widely acknowledged that a one-health approach, involving humans, animals and the environment, is needed to effectively address ABR [11–13]. In this context, environmental sampling of antibiotic residue and resistant bacteria is crucial for understanding the following processes: (1) the cross-transmission of resistant pathogens between human, animal and ecosystem, (2) the evolution of resistance in pathogens through pollution of bacteria and selective agents, (3) the impact of antibiotics on environmental health and (4) the surveillance of the prevalence of ABR in human or livestock and through environmental samples as well as (5) the use of antibiotics on a population level [14]. A recent joint technical brief from the WHO, the Food and Agriculture Organization (FAO), and the World Organization for Animal Health (OIE) highlights the importance of environmentally based surveillance in the global management of the ABR challenge [15].

The fourth objective overlaps with that of clinical surveillance. Hence, ABR surveillance based on environmental sampling has been proposed as an approach to bypass some of the challenges involved in clinical surveillance systems. In particular, the surveillance of sewage can provide information about enteric bacteria of several thousand people using fewer resources [16–22].

Regardless of the specific objective, environmental surveillance of ABR could add important knowledge for the protection of health and health systems, particularly in countries where antibiotic consumption and traditional clinical surveillance data are lacking [14]. According to a report from the Infectious Diseases Data Observatory, the more complex the sampling method for ABR surveillance is, the less likely it will be implemented into routine protocols, especially for LMI-settings [23] Examples from India show that ABR surveillance data are available from a few individual hospitals and a laboratory network, thereby limiting the national picture [24]. A study from Ibrahim and colleagues of clinical surveillance in Ethiopia describes several challenges that were faced in the early implementation stage, technical as well as social [25], and so do Bordier and colleagues in a report of the implementation of a national surveillance system in Vietnam [26]. Likewise, a recent review by Gandra and colleagues of eight Asian countries point to multiple challenges across social and technical dimensions for effective ABR surveillance [27]. Thus, the idea of simpler and affordable ABR surveillance methods is an attractive prospect for LMI-settings, which is why environmental surveillance approaches could be considered more feasible. If successful, it could both promote local ABR management and related health needs, and attend to the epistemic injustice created by local lack of information for these purposes in LMI settings and the global ABR information bias created by the lack of LMI data in GLASS [9].

However, there are many knowledge gaps when it comes to adequate environmental surveillance of ABR [17,28,29]. Depending on the objective(s), potential sampling sites include rivers, lakes, the ocean, drinking water or wastewater (treated or untreated). Additionally, samples of soil, sludge, sediment and solid waste may be used, depending on the objective. The practical process of reliable environmental sampling includes planning, practically as well as legally, and needs to be prepared by appropriate expertise in collaboration with local partners. Choice of type of sampling, identification target pathogens or compound, preparation for possible interferences (changing environmental conditions), location of sampling site, required equipment, requirement for sample preservation and permits, are just a few factors to be considered [30]. In addition, the fact that relevant experts are typically not from an LMI-setting could add to institutional and cultural challenges already known to exist. These latter types of factors have been highlighted in social science research on ABR [31–33], especially so regarding global and LMI-settings, highlighting how the feasibility and acceptability of standard measures from a high-income setting may be seriously affected by contextual differences brought by the move to an LMI-type situation [32,34–37]. As mentioned, the idea of an environmental approach to clinical and general ABR surveillance is primarily a response to some of such known challenges. But despite its basic importance for other aspects of ABR policy, the particular challenges of surveillance in LMI-settings have not so far been a focus in social science ABR research.

Currently, there is a development of research of environmental science and technology in this area [20,29]. Numerous recent studies have applied environmental sampling for ABR surveillance purposes in LMI settings [38–44]. The *Global Sewage Project*, which has coordinated sampling of municipal sewage for genetic antibiotic resistance analyses in more than 100 countries is probably the most extensive effort to date [45,46]. Also, research done within the framework of the WHO-initiated *Tricycle AMR surveillance project* represent development in this area [47,48]. Some of the LMI-oriented social science research mentioned earlier has touched on environmental dimensions of identifying ABR drivers and hotspots, and antibiotic stewardship, lifting, for instance, pharmaceutical pollution, infrastructural challenges due to economic limitations and a need for better integration of local collaborators [49–53]. However, as the particular challenges of surveillance have not been lifted in this research, nor has the specific notion of *environmental* approaches to overcome known feasibility challenges to effective ABR surveillance in LMI settings. A recent contribution from global development implementation science has presented a general framework for moving ABR-interventions from general idea and technical concept to effective implementation in a global health context, not least LMI settings [54]. The first step recommended is to ‘Conduct cross-sectional qualitative (key-informant interviews) … ’ [54, Table 1]. In the present case, due to the lack of previous charting of this particular technical solution, we found it desirable to start with systematizing perceived challenges to effective implementation from the point of view of parties that (a) understand the technology well and (b) have experience of applying it in the intended circumstances.

**Table 1.** Recruited key informants in terms of geographical origin, technical competence and LMI-related area of practical experience of ABR-related environmental surveillance.

Origin	Function	Experience from
Africa (1)	Environmental scientist	Africa
North America (1)	Environmental engineer	North America, Asia
Asia (1)	Environmental scientist	Africa, Asia
Europe (4)	Environmental chemist Environmental scientist Bioinformatician/Statistician Microbiologist	Europe, Africa, Asia

In this paper, we thus attempt a first step to enhance the understanding of barriers and pathways for ABR-related environmental surveillance in LMI settings. Based on interviews with experts on environmental ABR surveillance with relevant experience from LMI settings, we explore three main research questions:

1. What typical barriers for effective environmental ABR surveillance do the expert encounter in LMI settings, and what typical pathways are used by these experts to circumvent such barriers?
2. How do the experts reason around how these barriers arise, and how they relate to the pathways used?
3. How well do the overall patterns of identifying barriers and choosing pathways align with each other, as well as match the overall aim and rationale behind environmental ABR surveillance in LMI-settings?

Since the results reveal a number of inconsistencies and misalignments that may undermine both local and global aims of ABR surveillance, we then discuss how such risks may be overcome, and suggest a number of further actions by researchers as well as global institutional actors to improve the situation.

## Methods

### Design

The lack of earlier scientific coverage of the topic of this paper led to the choice of an exploratory qualitative interview study of key informants as a first step to start charting the territory. The data thus collected were analyzed using a thematic analysis approach [55,56], where main and sub-themes of relevance to the topic of the study and the practical context of its importance were generated to answer the research questions.

### Sampling

The methodological basis of the present study is the interview of selected *key informants* [57], i.e. informants already known to possess specialized knowledge of relevance to a study topic. The approach of using key informants was selected since no previous study of this particular topic exists that could inform how to design a more random or wider sampling, and it was thus important to find people who could be assumed to have the relevant experience to be able to inform a seminal analysis of the study topic. Included were participants of both male and female genders, all with a doctoral degree in environmental engineering, microbiology, environmental science or related fields, and more than four years of practical experience in environmental ABR-related surveillance in at least one LMI area.

The sampling was conducted in several stages, mixing *convenience purposive sampling* and *snowball sampling*. Initially, three key informants from Europe known to have relevant experience for the study were approached. Additional potential key informants were identified based on information from the first three, and some of these responded with further suggestions from their networks (*snowball sampling*). This process was continued until data saturation, resulting in a total number of 7 interviewed key informants. See Table 1 for a generic description of the origin, type of expertise and experience of these informants.

### Data collection

Based on the available literature about the study topic, an interview guide was developed. The guide contained open-ended questions and a thematically organized set of follow-up questions about the key informant’s experience and opinions in the area of study (see Appendix 1).

Each interview lasted between 45 min to 60 minutes and was conducted via Skype or in person during the period December 2019 – March 2020. All interviews were recorded, and then transcribed verbatim to text. After each interview, a short memo was written which included observations of apparently significant statements and own thoughts, for use in the subsequent analysis.

### Data analysis

The initial steps of the analysis focused on the first two research questions, regarding what barriers and pathways there are, and how the informants perceived and reasoned around the nature and context of, as well as the links between these barriers and pathways.

In the first round of this analysis, transcripts of the three initial interviews and the linked memos were read. Repeated or otherwise apparently significant statements were coded using the software NVivo to develop an initial coding scheme. This scheme enabled the initiation of a low-level conceptual analysis that provides a fundament of thematic understanding for subsequent rounds of analyses [56,58]. After multiple rounds of comparing and analyzing the data, a total of 18 themes related to research questions 1 and 2 were coded. In the second round of analysis, the remaining four interviews were added to the analysis, and new themes relating to research questions 1 and 2 were added if a significant statement did not match existing codes. This pattern was continued until no further thematic coding ideas relating to questions 1 and 2 for the available data from all seven interviews could be added. This principle is an analytical counterpart to the notion of data saturation. After 8 months, the informants were approached again to provide feedback to the existing results so far. This provided reassurance of the themes relating to research questions 1 and 2, and effected only very minor adjustments of a few details.

The resulting thematic map consisted of seven main categories related to research question 1 - three main types of barriers and four types of pathways. Each of these main themes contain variants that revealed details relating to research question 2, which we analyzed as a total of twenty-two sub-themes to the main themes. Based on this, we then moved the analysis to address the third research question. Here, the analysis shifted from a mainly inductive teasing out of thematic patterns and details in the observations made by individual respondents to a more abductive search for themes. This as research question 3 moved the analysis to a meta-level, where the entirety of the analysis relating to questions 1 and 2 was matched against the aims and rationales of environmental ABR surveillance in LMI settings, and standards of coherence. This analysis resulted in three main themes, with a total of nine sub-themes.

### Ethics

This study is not eligible for legally mandated ethical review according to the existing Swedish regulation and system for research ethical review [59]. We nevertheless applied standard procedures of informed consent to ensure respect for the integrity and autonomy of the study participants. All study participants were informed in writing about the aim and nature of the study and gave informed consent to participate in the study as well as to having the interviews recorded. All data (the interview recordings as well as pseudonymized transcripts) are stored in a secure way at the University of Gothenburg and can be accessed via contact with the research team. We also identified two risks not covered by Swedish research ethical review regulation, namely that overly specific identification of geographical regions in the publication of results could create social stigma of others than the informants (namely people living in these regions) and, additionally, impede future access of experts to these regions to conduct ABR-related environmental sampling in the future. These latter risks have been managed by making the information about geographical regions less specific in the overview table regarding the informants, as well as the interview outtakes used in the result section. In the interview quotes, such specific details have been replaced by generic terms in brackets.

## Results

**Figure 1.**
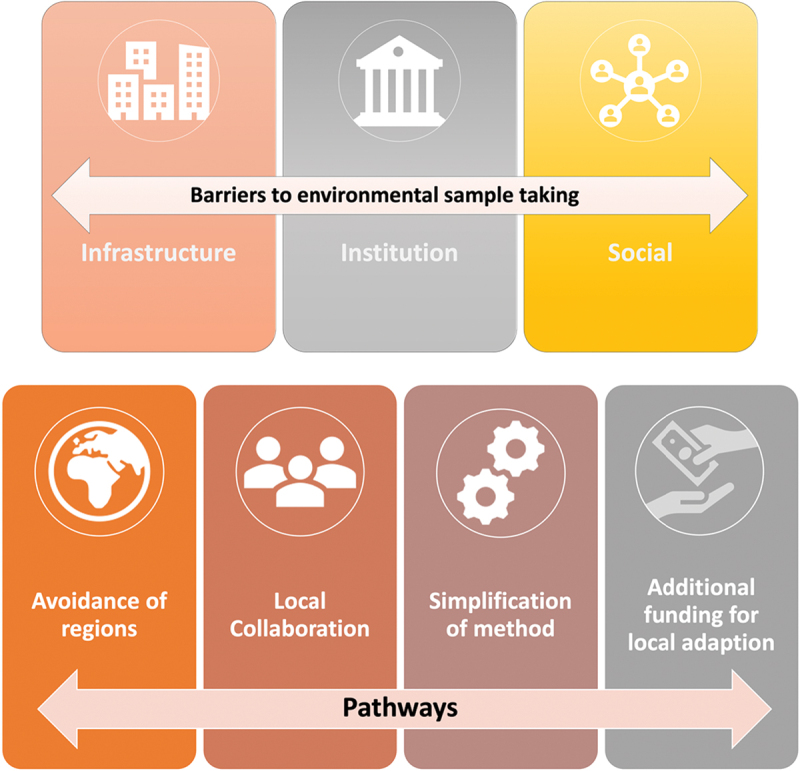
Thematic categories of reported barriers for ABR-related environmental surveillance, and pathways to overcome these barriers.

We found seven main themes that relate to research question 1, three under the main heading of barriers (infrastructural, institutional and social) and four under the main heading of pathways (avoidance of regions, local collaboration, simplification of methods, additional funding for local adaption), See Figure 1 for an overview.

The specifics and importance of these themes are explained below, with the help of examples from the data, and the overview tables for the sub-themes analyzed within each main category of barriers and pathways respectively, which relate to research question 2.

### Barriers

#### Infrastructure

Infrastructural barriers regard concrete physical or material hurdles to the effective implementation of surveillance methods. Three main types of such hurdles were described, overviewed in Table 2.

**Table 2.** Overview of reported infrastructural barriers to environmental ABR surveillance in LMI settings.

Infrastructural barriers		
Lack of organized infrastructure makes representative sampling infeasible	Unreliable local equipment and facilities prevent continuous sampling and adequate analysis	Design or maintenance of physical structures prevent access to specific optimal sampling spots
Due to, e.g. very decentralized sewage and wastewater infrastructure	Due to, e.g. electricity shortage, lack of machinery, suitable labs	Due to practical infeasibility or physical danger related to risk of falling or toxic contamination

Infrastructural barriers were described as especially challenging in rural areas, due to the condition of roads, unreliable sources of electricity and inappropriate equipment in local laboratories. The exact nature of the challenge depends on what kind of sample is aimed for, e.g. water samples from wastewater treatment plants (WWTP), effluent from hospitals, or soil from farmland.

The sanitation system could make it impossible to collect *representative* samples. In LMI areas, households are often not connected to a central sanitation system, but use locally diverse sanitation, like neighborhood-specific sewers (in cities), underground pit latrines shared by only a few households or open defecation:

The whole idea here is that it should also be suitable. It should require less resources due to that we can basically just go to one sampling place, one sampling point, take one sample and get the information. If we instead would have needed to go to thousands of pit latrines than we have not earned that much, actually.

[…] and then you also have something called open defecation. People that don’t use pit- latrines. And then you can have the combination of human gut flora and high levels of antibiotics in the river system. That also much trickier to sample because you have to know so much about the customs of the country and the particular city.

Facilities often lack automatic sampling equipment, or laboratories are unreliable. This impedes both continuous sample taking and limits what analyses of samples can be undertaken:

Ideally, you would like to have a sampling device that could take flow-proportional sampling as well and automated sampling but that is usually not the case.

Sampling is not that difficult. But then if you want to do some kind of pretreatment of the samples or store them in a freezer. Even a freezer may be kind of not available, or if they think that there is a freezer, it may not be functional or something like that. So the infrastructure in these countries is very underdeveloped.

A final aspect of infrastructure is that access to otherwise suitable sampling spots are too difficult or overly dangerous to access:

We had said for all the plants we’re going to sample at a certain point, but at that specific plant, that point was quite dangerous to access. It’s not that they didn’t want us to, but we just couldn’t […] There’s another aspect that could be an issue, but I’m not sure and that would be potential infectious diseases that might be riskier around or within the wastewater treatment plants themselves.

#### Institutional barriers

Institutional barriers regard impediments to implementing effective environmental ABR surveillance methods due to systems of law, political or managerial governance or public administration, including their real-world operations. Four main types of such barriers were described, overviewed in Table 3.

**Table 3.** Overview of reported institutional barriers to environmental ABR surveillance in LMI settings.

Institutional barriers			
Political governance	Business and managerial interests and structures	Bureaucracy	Political and managerial priorities
Authoritarian political governance tends to impede access to sites and resources more	Due to, e.g. corruption, power relations or career-related interests	Due to difficulties of securing adequate collaboration agreements, and permissions and authorizations for sampling, analysis and international transfer of samples.	Environmental ABR-related concerns low on the agenda, often due to limited economic circumstances

The political governance structure of a state could influence access to sample taking and laboratory facilities. All informants mentioned that authoritarian governance, the extreme being outright totalitarian states, tended to worsen such impediments. One reason for this raised by the informants was that authoritarian rulers are less open to activities that might reveal negative aspects in their country or region:

If the government is a dictatorship and has the idea about their image or their appearance would be diminished or affected somehow by the results.

Ownership and management structures of facilities of interest, such as labs, WWTPs or farms, was also mentioned as potential barriers:

You have a supervisor who has no control over the work of the plant manager, because the supervisor cannot query the plant manager or hold the plant manager accountable because he was employed by somebody higher up than the supervisor whose employment was not based on merit, but on the fact that he knows somebody.

Bureaucratic obstacles, ranging from securing formalized collaboration agreements in the form of so-called memoranda of understanding (MOU) [60] to obtaining ethical permits, were described as more cumbersome and slower in LMI areas than in high-income countries familiar to the informants:

You need to specify exactly what you should do with the sample. They would like to be informed about the results. Also, what we will write about the results before we publish them. And in some cases, they also want us at least in one particular case, I remember they would like us to keep the treatment plant anonymous, no name, no exact location.

Some partners required that we have a memorandum of understanding (MOU) formal agreement in place before we start working. Even a research agreement that is just between one researcher to the other doesn’t work in some instances, so they want the universities to have an MOU stipulating the nature of collaboration and all that in place before we start working. So usually delays will start from here in a sense of getting the approval from my university, and then also getting the approval from the partner university as well.

Ethical permits related to the Nagoya protocol of biodiversity [61], which regulate access and benefit sharing of natural resources for sample taking and subsequent handling of pathogens, were held out as linked to strong local concerns of undue exploitation of natural resources without benefit-sharing:

The major concern of the ethical part has been that you’re taking wastewater and you’re looking for, let’s say pathogens, because we’re looking for antibiotic resistant e. coli which we assume is pathogenic. So, the ethical concern is that you are taking the wastewater, that is from people and it’s going to be identified by a location. So, people have been concerned that you are taking their wastewater.

Institutional barriers could also be worsened by strained local finances, pushing the environmental surveillance aspect back in the line of ABR-related priorities:

So, if you’re having a problem with antibiotic resistant bacteria, killing a lot of people in the hospital and they have, 1000 euros, then I wouldn’t expect them to say what’s the situation like in water or soil or whatever. First, I would like them to use that money to look at the patient’s themselves.

#### Socio-cultural barriers

This main category entails all socio-cultural aspects that are not institutional, such as language, custom, religion, ethos, and social (in)stability. Five types of such aspects were reported to impede effective surveillance, overviewed in Table 4.

**Table 4.** Overview of reported socio-cultural barriers to environmental ABR surveillance in LMI settings.

Socio-cultural barriers				
Working culture	Knowledge and know-how	Language and communication	Corruption and sharing ethos	War and social unrest
Experts hold different expectations of performance and autonomy of local collaborators than local collaboratrs themselves	Implementation of surveillance methods impeded by lack of local knowledge and skill, and experts lack of understanding of local contexts, enhancing all other institutional and social barriers.	Language differences, related translation problems and different communication ideals impede implementation and enhance institutional and other socio-cultural barriers.	Differences between surveillance experts’ views of what is expected and unacceptable and that of local collaborators impede collaboration and enhance institutional barriers.	Unstable sociopolitical situation or outright war-like circumstances impede experts to supervise on site (due to safety issues or bans to enter areas) or to form local collaborations.

These different sub-categories overlap and interlink in many ways. Below are selected quotes to illustrate the specific sub-themes, as well as commentary to highlight how the sub-themes interlink. Overall, it may be observed that the socio-cultural theme is quite broad, including both hard demographic aspects and soft culture in the form of informal social expectations.

Working culture was highlighted in several ways, both as blunt observations of differences of expectations impeding activities through misunderstandings, and as something that by itself undermines the experts’ ability to control surveillance activities and execute them as planned:

when I tell somebody to do the sampling, they know I just want a sample. So maybe they just pour the one liter and they leave, although I may have given them instructions on how to sample, but there’s no way to supervise and ensure that they actually did it the way you wanted it to be done.

In [Asian state], it was my postdoc who was able to communicate with the [local collaborators] and guide them to do these things and so on. So that was much more efficient. […] But now in Africa, I don’t have anybody in my group who would be the local, which means that it’s much more inefficient and much slower. So in practice nothing really happens unless somebody from my group is there.

This second quote also touches on the second sub-theme, as it describes a situation where an external instructor needs to be added due to lack of local understanding of what to do and how to do things. Another informant described how such knowledge differences also could create atmospheres of distrust and fear:

The people trying to protect themselves. If they think that you uncover something that is not the right way what they should be doing. Like in our treatment plans, for instance, we go there and realize that the operators may not be skilled enough to be operating a plant, but they are there.

Language differences and risks of misunderstanding were implicit in several socio-cultural sub-themes, both the preceding ones and ones following below. However, the informants also described specific language-related differences of general communicative culture (rather than syntax and grammar), such as how to ask a question in a way that is likely to elicit a true answer:

When we asked: do you give antibiotics to animals? And the farmer said, No. Then my postdoc thought that maybe I should ask in a different way. Maybe I should ask what are you giving to the animals. And then they were willing to show. No, they didn’t want to hide anything. So we found out that they were giving antibiotics in a prophylactic way.

Several examples were given of how the experts had experienced a local culture of corruption they had problems adjusting to:

It’s difficult for them to see their role because they want to be kind of it would like to be kind of like partners, that we are on the same level of the project. But on the other hand, they are expecting us to tell them what they should do, and also to fund everything. So that’s kind of a bit contradictory.

At this research institute, things went very slowly at the beginning. It took about half a year for us to understand that they want to be asked to pay them, like a bribe, which they even say openly, but then we understood it. And we didn’t want to do that

At the same time, the informants also described that they themselves were not very forthcoming to share the potential benefits of projects with local partners, and that this may undermine necessary collaboration and create longstanding distrust.

I noticed that the perception of the professor was that: Okay, he is coming for a project and externally funded projects, so there’s money involved. So I’m there and I realized that okay, he invited me to visit, but upon our first discussion, when he realized that this is no what I am offering, then he actually was no longer interested in my stay anymore.

There are instances that they give you the chance to do the study and then at the end, they don’t have the data. They end up reading about it in a publication or at a conference and something of that nature. So there is a bit of mistrust between the collaborators as well.

The final sub-theme here includes the more violent aspects of differences in socio-cultural contexts, which could make surveillance projects very difficult to undertake. While the first example here regards war-related hurdles, the second one is more about how local corruption may create social hostility and unrest.

I have a collaboration with a researcher that lives in the middle part of [African state] and the [University] department has explicitly said that it’s bad to go to that part. It’s [militant group] country.

The third time was a bit more hostile, but that was because what we were measuring was what affected the image of the individual company. And especially when they had this illegal dumping grounds and that was actually polluted pretty close to the facility. There were some hostile words. People were shouting and stuff like that.

### Pathways to address the barriers

#### Avoidance of regions

Avoidance of regions means that an expert on environmental AMR surveillance who perceives barriers to such surveillance in a region manages these barriers by simply avoiding to survey this region. This pathway to deal with barriers was reported across all informants, but varied slightly regarding the grounds in the form of perception of barriers. Three different grounds were described in this respect, overviewed in Table 5.

**Table 5.** Overview of reported grounds to avoid regions due to barriers.

Avoidance of Regions		
Earlier (discouraging) experience of region	Warnings about region	Known complications in regions
The expert has tried to conduct environmental AMR surveillance in a region, and been discouraged from trying again	The expert has received intelligence about barriers that discourages from attempts at environmental AMR surveillance	The expert has foreknowledge of barriers in a region that discourages from attempts at environmental AMR surveillance in this region

The general theme, as well as the mentioned grounds are illustrated by the following reports from the informants:

You tend to know which countries you should avoid or where you can expect problems.

Researchers are, I wouldn’t say lazy but if you study soil, it’s easier to just go and dig here instead of going around the world to start it somewhere else.

There are urban areas, with no sewage systems. So there are cities with more than half of the population not connected to any sewage system. We don’t even consider taking samples in rural areas because urban is not even really connected.

#### Simplification of methods

This pathway around barriers to environmental ABR surveillance consists in adapting to barriers that impede the feasible implementation of optimal methods by simplifying these methods to the point where they are feasible in spite of the barriers. Two variants of this strategy were described, overviewed in Table 6.

**Table 6.** Overview of reported ways to simplify methods due to barriers.

Simplification of Methods	
Managing unforeseen problems by creating an alternative, sub-optimal, methodological solution on the spot.	Planning that adapts all methods to fit local conditions before implementation
Such as sampling “as close as possible” when the optimal sampling location cannot be accessed	Trying to foresee barriers that make optimal methods infeasible, and adapt the implemented methods

Each of the quotes below illustrate one of these variants.

We didn’t take samples at individual factories and facilities, but we tried to take them as close as possible.

We have to think about the best solution for a certain condition and obviously solutions that are feasible in [Europe] won’t be feasible in [Africa]

#### Local collaboration

This theme captures the strategy to find ways to circumvent barriers with the help of local collaborators. This pathway could be varied situationally and be more or less available, depending on type of barrier faced, the local context and the way of initiating collaboration. We have analyzed five main variations in this respect, overviewed in Table 7.

**Table 7.** Overview of reported roles for collaborators and ways of securing them.

Local Collaboration				
To manage language problems	To adjust logistics	To navigate administrative and legal barriers	Initiated by chance	Initiated by trustful relationships
A local natural language speaker acts as interpreter and translator	Someone with local knowledge about infrastructure, technical equipment, transport, etc. helps with information and solutions to manage related barriers	A local resident or knowledgeable longtime visitor helps with procedural advice and introductions to adequate administrators	Luck provides a suitable collaborator that just happens to be able to assist	Relationships can be built through local capacity building for staff, openness regarding aims of the expert and related benefit sharing

The point of having a local collaborator was described as a help with circumventing barriers of different kinds: to adjust logistics or initial plans, to handle institutional or social or language problems. Mostly this collaborator was described as a local resident, but local diplomatic staff from the country of the visiting expert that know how to deal with local administrative difficulties was also mentioned as a possibility:

We were discussing about this bureaucracy, and she said that well, you may also have just a scientific seminar with the locals, and then part of the seminar is to take samples and the locals can take the sample and then you don’t need any permit. So even the ambassador taught us how it would be easy to circumvent the need to have a permit.

I wouldn’t be able to go there to take samples myself, but you can bypass that by working together with local authorities and local researchers.

I had to use his student who also needed assistance from me to do parasite analysis. So then we went for the sampling so that I did the parasite analysis for the student, after which I could do my analysis for antimicrobial resistance. To have the access that I needed.

At the same time, although this was held out as being of crucial importance, no method for securing truly *suitable* local collaborators was mentioned. Finding one mostly appeared as an outcome of luck. However, the interview question gave rise to some speculative reflection that local collaborators might need to become more involved to provide suitable help, e.g. granting access to sample-sites:

So, in as much as we can see for the treatment plants is the operators that are not willing to cooperate, sometimes we are also not sincere and open with how we interact and sharing data with them.

Having a really local partner is not essential of course but it’s even better if you have a partner that you really know. For example, some local, who has been in your lab, even got the degree in your lab and then goes back to the native country. That makes things much easier.

Gaining the confidence of the wastewater treatment plant controllers or operators, and, dealing with them in terms of sampling and devising protocols of how to sample already helps.

#### Additional funding for local adaption

One possible way to deal with financial limits enhancing institutional or social barriers that was mentioned speculatively was to help secure external special funding to facilitate the local prioritization of the environmental surveillance aspect of ABR, which might help with mitigating or managing several of the barriers.

So again, that’s something regarding funding that either there has to be external funding from agencies that specifically fund that or we have to realize that it’s not fair to tell the farmer don’t use antibiotics if he doesn’t have a solution.

## Overall alignment of the themes and sub-themes

Analyzing the themes and sub-themes relating to research questions 1 and 2 taken together resulted in three main meta-themes and eight sub-themes relating to research question 3. These are overviewed in Tables 8–10, with explanatory examples following each main theme.

**Table 8.** Overview of how chosen pathways undermine aims of environmental ABR surveillance.

Counterproductive pathway choices		
Avoiding regions and sites goes against the core aim of LMI environmental ABR surveillance	Adjusting methods threatens to undermine the core aim of LMI environmental ABR surveillance	Both these pathway choices threaten to maintain or worsen ABR-related global epistemic injustices
The strategy maintains both the gaps in global ABR surveillance and the local lack of surveillance data that the environmental approach is supposed improve.	The strategy undermines the chance of filling gaps in global ABR surveillance and addressing local needs of surveillance data that the environmental approach is supposed improve.	Addressing the global epistemic injustice created by lack of reliable ABR surveillance data from LMI settings is a further main rationale for addressing the lack of such surveillance data, besides global health promotion.

**Table 9.** Overview of how some pathways fail to adequately address barriers.

Misaligned barriers and pathways		
Lack of systematic strategy for establishing local collaboration	Lack of strategy to make collaborators benefitting partners	Lack of strategy for local capacity building
Despite local collaborators pointed to as primary way to manage barriers without avoiding regions	Despite observation that benefit sharing could aid in establishing trustful local collaboration	Despite observation that local capacity building could aid in establishing trustful local collaboration

**Table 10.** Overview of sense of disempowerment interplay with structiural factors.

Structurally embedded self-inefficacy		
Unmotivated sense of powerlessness in the face of barriers	Structural factors do limit feasible options of researchers and experts	Researchers and experts could initiate structural action and reform
Researchers and experts could probably do more to overcome hurdles for productive pathways around barriers than what they project themselves	Several structural factors, from war to existing socio-economic contexts, are out of reach for researchers and experts to influence.	Institutional global actors within and outside the research community could be prompted to take action to prevent barriers and better support researchers and experts to implement more productive pathways.

While the avoidance of sites or regions is held out by the respondents as a main pathway to overcome barriers, this strategy at the same time serves to maintain existing knowledge gaps regarding the ABR in LMI settings. Completely avoiding an area leaves the map of the ABR problem that is crucial for effective action blank in this area. At the same time, recent environmental monitoring efforts suggest that it is often in those countries that largely lack clinical surveillance that have the most worrisome antibiotic resistance situation [45,62]. Locally, crucial information for effective clinical ABR management adapted to the local conditions thereby remain scant.

The pathway of simplifying methods unfortunately adds to this counterproductivity, as less perfect surveillance methods limit the reliability and range of the collected data. In effect, even if some additional data is retrieved through this pathway, it will be difficult to trust and leave significant uncertainties.

Combined, these upshots not only undermine effective ABR management in LMI settings and create an inequality of prospects to handle the ABR challenge between LMI and other areas. This inequality additionally enhances the already existing epistemic injustices, mentioned at the outset as a main reason to mind more about the conditions for environmental ABR surveillance in LMI settings.

The second theme is not about lack of alignment with external aims and rationales, but rather about internal incoherence between the informants’ own perceptions of the barriers and their reported choices of pathways. Earlier it was pointed out and illustrated how the informants held out local collaboration as a perceived key strategy for overcoming many barriers, but nevertheless seemed mostly to leave the securing of such collaborators to chance. In view of the foregoing theme of the counterproductivity of main pathways, this creates a lack of fit between the informants’ own views on what would be a fruitful way to manage barriers, and their account of their own choice of pathways. Of course, there may be many explanations for why more focused efforts to secure local collaboration are not lifted by the respondents, and we will expand on that point below. But the lack of fit as such nevertheless expands Sariola and colleagues’ [49] observation of the importance of good strategies for securing local collaboration in LMI settings for ABR research and stewardship into the environmental surveillance domain. One particularly striking aspect is how respondents from a well-funded high-income context underline that potential local collaborators may express interests in sharing benefits (such as resources and research merits), while these informants report themselves not to consider this and even to hide further agendas (such as research publication or expert reporting), in which collaborators might benefit from being included. A specific type of benefit sharing to secure local collaboration would be for experts with available funding opportunities to do so to design projects to include local capacity building. This could be in the form of technical resources (e.g. to improve local lab capacity – by itself an identified barrier), or even in the form of training local staff, rather than ‘helicoptering in’ outside, well-funded expertise that leaves once the project is finished. Both these examples are in line with general calls for better equity and inclusion in global health research [63]. This observation leads over to the last main theme.

Self-efficacy is a psychological notion denoting a person’s own sense of ability to influence one’s situation and attain one’s goals [64]. Our overall impression from the themes in this and foregoing subsections is that the respondents express major *in*efficacy, i.e. a lack of sense of ability with regard to the barriers. This is to a large extent understandable, given the complexity of the combined barriers. First, some experts operate out of LMI-contexts and may suffer from resource scarcity themselves, thereby being prevented from including much of local benefit sharing or capacity building in their own projects. But even those well-funded experts that operate out of high-income areas will be unable to change major infrastructural barriers or security threats (such as war). Moreover, a small outside player, like a single expert team, is not normally equipped to deal strategically with the sort of multifaceted, partly structural challenges created by several variants of the social and infrastructural barriers. The reported response of mostly ‘muddling through’ or avoid, with the latter as the mostly preferred option, may in this light be expected. Existing regulation, administrative requirements and social customs are as they are, while a visiting expert is normally legally prevented from handing out bribes.

At the same time, in some respects, the sense of disempowerment reported by respondents seems to us to create an overly paralyzing effect on attempts to improve on the status quo. This is most obvious regarding the challenge of securing suitable local collaboration, explored in the foregoing main theme. Surely, experts from high-income situations could organize more systematic explorative inquiry to find workable ways to secure suitable local collaborators to overcome different types of barriers in different settings. Such work could proceed using formats of surveys, focus groups or workshops, where representatives of experts and local stakeholders come together to generate new ideas and, at the same time, be mobilized to attend to observed problems related to environmental ABR surveillance [65]. Action-oriented social science researchers in the global health area could help facilitate such endeavors [49]. These experts could also put more effort into including capacity building into the funding of projects from the very start. Finally, all experts (also those with a more limited funding context) could include potential local collaborators as *scientific* co-planners and potential beneficiaries of *scientific credit*.

This being said, it is rather obvious that the structural nature of several of the barriers, and the complexity when they occur together, does mean that they are best addressed at a structural level. The respondents here lift the possibility of having development organizations and high-income state agencies support projects that would prevent or mitigate barriers (e.g. by improving relevant infrastructure). They also imply that such external support might facilitate potential collaborators to prioritize ABR surveillance development. To this, we may add the possibility of having relevant research funders, such as the WHO, the JPIAMR, the Wellcome Trust, the NIH or the Gates Foundation, more forcefully require and facilitate local benefit sharing and capacity building in the global health ABR-related projects they support. Experts with the standing to assert influence in this regard – individually or collectively, possibly via their own research community organizations – could here act to help initiate and implement these latter changes in funding calls, review and selection of projects.

## Discussion

The respondents report 3 main types of barriers (infrastructural, institutional and socio-cultural) for effective environmental ABR surveillance in LMI settings, and 4 main pathways to overcome these barriers (avoidance of regions, adjustment of methods, local collaboration and additional funding for local adaption). The general nature of the barriers align well with previous ABR social science research related to LMI settings overviewed in the background. The respondents’ perceptions of and reasoning around the barriers and pathways reveal a broad selection of variants of each type of barrier and pathway, creating a high degree of complexity faced by experts when undertaking attempts at environmental ABR surveillance in these settings. Securing good local collaboration stands out as the pathway viewed as most promising by the respondents. Also this result fits well with results from social science research on environmental ABR stewardship and research in LMI settings. At the same time, the respondents report no systematic efforts to secure collaboration and even some actions that undermine collaborative prospects. This creates an internal tension in the experts’ overall view of the barriers and pathways. In addition, the pathways chosen in the absence of local collaboration appear counterproductive relative to the rationale for environmental ABR surveillance in LMI settings. Overall, experts project weak self-efficacy regarding prospects of managing barriers in ways that would avoid the mentioned downsides. While this is understandable structural and complex nature of the barriers, it also sometimes seems overly disempowering, especially in relation to experts operating out of high-income contexts. These could probably include more of benefit sharing and capacity building to promote good local collaboration. At the same time, many barriers would be best addressed on a structural level, via development organizations and global research funders.

The upside of these observations is, of course, that there appears to be room for improvement of expert practices related to environmental ABR surveillance in LMI settings. We have held out some obvious low-hanging fruits in this regard. Experts from high-income settings could take many steps directly to better include local collaborators and capacity building, and adding on social science layers to projects could help facilitate such collaboration and optimize its outcome. Experts could, via their scientific community organizations, also lobby to have outside development organizations act to remove or reduce barriers altogether, or to help local actors to prioritize ABR-surveillance, thereby facilitating long-term improvement of global health and justice.

One especially available route for experts to address structural factors would be to appeal to their own academic community organizations and major research funders in the ABR and global health domains. At the same time, we want to underline the importance of two complementary sides of research practice and funding reform to incentivize better local collaboration. Surely, one side has to be stronger and more specific requirements of environmental ABR surveillance projects in LMI-settings to include local benefit sharing, capacity building and true partnership. However, all of that adds costs to projects, and if no other actions are taken by research organizations and funders than adding the stated requirements, the risk is obvious that experts are discouraged from instigating projects to improve ABR surveillance in LMI settings, To avoid thereby adding further counterproductivity and worsened injustice to what has already been highlighted, the reform therefore also has to include increased funds to cover the associated costs. In addition to being in general line with the mentioned calls for better inclusion of local partners in global health research [62], this would also address the specific inequities burdening the ABR-related parts of such research [10].

While this study is a mere first step in an implementation science process [54], its seminal nature serves to reveal substantive and important factors to consider when developing environmental ABR surveillance strategies for LMI settings, despite a relatively small sample size. This and its exploratory, qualitative approach, of course, bring some limitations, but none that weakens the analytic findings, grounds for concern and suggested actions mentioned earlier – especially since the informants do represent relevant experiences from many different LMI settings across the world. Additional studies built on results of this one could explore several of the themes more in-depth. Likewise, expanded samples (including more experts and other stakeholders) could serve to add important factors, details and depth to consider when acting on the problems lifted in the present study. Quantitative or mixed-method surveys could add relevant information on how common and widespread the various phenomena explored in the present study are. And, as mentioned, action oriented social science studies could be added onto more technically oriented environmental ABR surveillance projects to further explore and facilitate local collaboration and fruitful ways to handle barriers in LMI-settings.

## Conclusion

Environmental ABR surveillance has great potential value for overcoming known challenges in LMI countries and related injustices of global ABR monitoring. Still, key experts report several barriers for implementing effective environmental ARB surveillance in LMI settings. In addition, many pathways for avoiding or mitigating these barriers are either insufficiently applied or counterproductive from a global health and global justice standpoint. Critical discussion within the global ABR research and management community is called for regarding, e.g. standards for benefit sharing, capacity building and openness with local LMI country collaborators to build trust. Global actors, such as JPIAMR, WHO and environmental and development agencies have reason to consider actions to mitigate existing barriers and facilitate constructive pathways to manage them. Expanded qualitative social science research, as well as quantitative surveys addressing a broader global selection of informants, may assess further possible barriers and pathway types and how common these are.

## Appendix 1: Interview Guide

Intro: Even though antibiotic resistance is a very popular and important research area, we still lack information in certain fields. One fundamental knowledge gap is the problem of monitoring the prevalence of antibiotic-resistant bacteria in the environment. This study tries to explore the impediments of accessing locations for sample taking in and around hospitals, pharmaceutical manufacturing sites or WWTP’s for surveillance studies with a focus on low and middle-income settings.

1. Tell me a bit about your current activity in research and describe your special interest in surveillance of ABR.

     o Are you focussing on the general environment or rather specific places like inside and outside hospital, pharmaceutical manufacturing sites?

2. What are the main/typical locations for sample taking in hospitals and manufacturing sites?

        o Inside or outside? Surfaces or water?

3. What are the main accessibility problems that you are facing.?

        o How do you manage accessibility problems? E.g. if you are not allowed to take samples from inside a factory

         o Can you give examples of situations where the access was perceived difficult, but you were able to overcome it?

4. What do you think are the main reasons for public or private decision-makers to deny access?

5. Do the pathways of data collection differ between LMIC and HIC? If so, how?

6. What kind of differences do you observe between rural and urban areas in terms of access?
